# Dental conditions in inpatients with schizophrenia: A large-scale multi-site survey

**DOI:** 10.1186/1472-6831-12-32

**Published:** 2012-08-18

**Authors:** Hideaki Tani, Hiroyuki Uchida, Takefumi Suzuki, Yumi Shibuya, Hiroshi Shimanuki, Koichiro Watanabe, Ryosuke Den, Masahiko Nishimoto, Jinichi Hirano, Hiroyoshi Takeuchi, Shintaro Nio, Shinichiro Nakajima, Ryosuke Kitahata, Takashi Tsuboi, Kenichi Tsunoda, Toshiaki Kikuchi, Masaru Mimura

**Affiliations:** 1 Department of Neuropsychiatry, Keio University School of Medicine, 35 Shinanomachi, Shinjuku-ku, Tokyo 160-8582, Japan; 2 Geriatric Mental Health Program, Centre for Addiction and Mental Health, 1001 Queen Street West, Toronto, ON M6J 1 H4, Canada; 3 Department of Dentistry, Bright Dental Care, 1-65 Namikimotomachi, Kawaguchi-shi, Saitama, 332-0033, Japan; 4 Department of Dentistry, Sakuragaoka Memorial Hospital, 1-1-1 Renkouji, Tama-shi, Tokyo, 206-0021, Japan; 5 Department of Psychiatry, Ohizumi Hospital, 6-9-1 Ohizumigakuennchou, Nerima-ku, Tokyo, 178-0061, Japan; 6 Department of Psychiatry, Komagino Hospital, 273 Uratakaomachi, Hachiouji-shi, Tokyo, 193-8505, Japan; 7 Department of Psychiatry, Touyokokeiai Hospital, 4-17-23 Arima, Miyamae-ku, Kawasaki-shi, Kanagawa, 216-0003, Japan; 8 Department of Psychiatry, Tokyo Musashino Hospital, 4-11-11 Komone, Itabashi-ku, Tokyo, 173-0037, Japan; 9 Department of Psychiatry, Nakayama Hospital, 2-10-2 Nakayama, Ichikawa-shi, Chiba, 272-0813, Japan; 10 Department of Psychiatry, Minamihannou Hospital, 415 Yaoroshi, Hannou-shi, Saitama, 357-0042, Japan

**Keywords:** Aging, Dental caries, Schizophrenia, Smoking, Tooth brushing, Tremor

## Abstract

**Background:**

Clinical relevance of dental caries is often underestimated in patients with schizophrenia. The objective of this study was to examine dental caries and to identify clinical and demographic variables associated with poor dental condition in patients with schizophrenia.

**Methods:**

Inpatients with schizophrenia received a visual oral examination of their dental caries, using the decayed-missing-filled teeth (DMFT) index. This study was conducted in multiple sites in Japan, between October and December, 2010. A univariate general linear model was used to examine the effects of the following variables on the DMFT score: age, sex, smoking status, daily intake of sweets, dry mouth, frequency of daily tooth brushing, tremor, the Clinical Global Impression-Schizophrenia Overall severity score, and the Cumulative Illness Rating Scale for Geriatrics score.

**Results:**

523 patients were included in this study (mean ± SD age = 55.6 ± 13.4 years; 297 men). A univariate general linear model showed significant effects of age group, smoking, frequency of daily tooth brushing, and tremor (all p’s < 0.001) on the DMFT score (Corrected Model: F_(23, 483)_ = 3.55, p < 0.001, R^2^ = 0.42) . In other words, older age, smoking, tremor burden, and less frequent tooth brushing were associated with a greater DMFT score.

**Conclusions:**

Given that poor dental condition has been related with an increased risk of physical co-morbidities, physicians should be aware of patients’ dental status, especially for aged smoking patients with schizophrenia. Furthermore, for schizophrenia patients who do not regularly brush their teeth or who exhibit tremor, it may be advisable for caregivers to encourage and help them to perform tooth brushing more frequently.

## Background

Patients with schizophrenia have been reported to suffer a variety of physical co-morbidities, which is considered to be attributable to their sedentary life-style [1] and impairment in self-care [2] as well as the side effects from psychotropic medications [3]. In light of the chronic nature of this illness, identifying and managing these physical conditions is critically important in this population. Among these conditions, the clinical relevance of dental caries is often underestimated [4] while other somatic conditions such as hypertension, diabetes mellitus and osteoporosis have received wide attention [3,5-7]. In fact, better dental conditions are known to be associated with not only an enhanced quality of life for patients [8], but also better digestion [9]. Previous surveys have demonstrated that patients with schizophrenia visit dentists less frequently, compared to healthy people, because of their difficult financial conditions and a lack of motivation in the maintenance of dental hygiene due to the illness [10-12]. However, while several previous surveys have examined demographic and clinical characteristics that are related with worse dental hygiene in schizophrenia [13-17], such data are still limited in the literature. Moreover, dental hygiene is expected to be subject to direct and indirect influences of current and local standards of care, which indicates a necessity of further information from various clinical settings in order to provide a robust agreement on this issue.

Therefore, in the present study, we conducted a cross-sectional study to identify variables associated with dental caries in a larger sample of persons with schizophrenia in Japan.

## Methods

### Study population

In this cross-sectional study, subjects were eligible if they were 18 years of age or older and inpatients and were diagnosed with schizophrenia or a related psychotic disorder (F20 - F29 according to the ICD, 10^th^ edition) [18]. We approached potential subjects in the units whose consultant psychiatrists agreed to the present survey. This study was conducted in compliance with the Helsinki Declaration at various hospital sites in Japan - Sakuragaoka Memorial Hospital, Musashino Hospital, Komagino Hospital, and Ohizumi Hospital in Tokyo; Nakayama Hospital and Kinosaki Hospital in Chiba; Minamihannou Hospital in Saitama; and Touyokokeiai Hospital in Kanagawa between October and December, 2010. All of these sites were psychiatric hospitals that were affiliated with either Keio University or St. Marianna University. They were located in the greater Tokyo area and had 200–700 inpatient beds. The study was approved by the institutional review board at each participating site, and subjects provided written informed consent after receiving detailed information about the protocol.

### Assessment of dental status and clinical and demographic variables

Patients’ dental caries were examined by visual oral examination by one of the investigators (HT or HS), using the Decayed-Missing-Filled Teeth (DMFT) score. The DMFT score is a standard index to evaluate dental caries recommended by the World Health Organization. This score ranges between 0 and 28; a higher DMFT score indicates a patient’s poor dental condition [19]. Symptomatology of schizophrenia and physical complications were assessed with the Clinical Global Impression-Schizophrenia (CGI-SCH) [20] and the Cumulative Illness Rating Scale for Geriatrics (CIRS-G) [21], respectively, by their psychiatrist of record. Other information collected included: age, sex, duration of illness, smoking habit, frequency of tooth brushing per day, presence of daily intake of sweets, presence of dry mouth, tremor by using the Udvalg for Kliniske Undersøgelser (UKU) Side Effect Rating Scale [22], and psychotropic medications prescribed.

### Statistical analyses

Statistical analyses were carried out with the SPSS Version 17.0 (SPSS Inc., Chicago). A univariate general linear model was used to examine the effects of the following variables on the DMFT score: age group (i.e., <40 [young], 40–59 [middle-aged] or ≥60 [old]), sex, smoking (i.e., smoking or non-smoking), daily intake of sweets, dry mouth, frequency of tooth brushing per day (< once/day, once/day, twice/day, and ≥ three times/day), the UKU tremor subscale score (0–3), the CGI-SCH Overall severity score (1–7), and the CIRG-S total score (<5 or ≥5). This model was generated with main effects and all significant 2-way interaction terms. The general linear model incorporates a number of different statistical models, including analysis-of-variance (ANOVA) and multiple regression analysis. When the univariate general linear model showed a significant effect of any subgroup/category-of-interest on the DMFT index, we also examined group differences with pairwise comparisons, using Tukey-Kramer HSD (honestly significant difference). A p-value <0.05 was considered statistically significant and all tests were two-tailed.

## Results

### Characteristics of the sample

Of the 550 inpatients approached for participation in this study, 523 patients (95.1 %) agreed to participate and completed all the study procedures. Demographic and clinical characteristics are summarized in Table1. In addition, psychiatric diagnoses of the subjects were as follows: schizophrenia (n = 511), schizoaffective disorder (n = 5), delusional disorder (n = 6) and acute and transient psychotic disorder (n = 1). The five most frequently prescribed antipsychotic drugs were risperidone (n = 194), olanzapine (n = 105), haloperidol (n = 58), quetiapine (n = 33), and blonanserin (n = 32). 202 subjects (39.0 %) were receiving more than one antipsychotic drug (Table1).

**Table 1:** Demographic and clinical characteristics of 523 subjects

**Characteristics**	**Values**
Age (years), mean ± SD (range)	55.6 ± 13.4 (18–87)
< 40 years, n (%)	75 (14.3 %)
41–59 years, n (%)	221 (42.3 %)
≥ 60 years, n (%)	227 (43.4 %)
Male, n (%)	297 (56.8 %)
Duration of illness, mean ± SD (range)	29.8 ± 13.9 (0–60)
GAF, mean ± SD (range)	33.3 ± 12.4 (5–85)
CGI-SCH, Overall severity, mean ± SD (range)	4.6 ± 1.0 (2–7)
CIRS-G total score, mean ± SD (range)	5.5 ± 3.1 (0–20)

SD, standard deviation.

### Clinical and demographic characteristics associated with dental caries

A univariate general linear model demonstrated that the following terms had significant effects on the DMFT score (Corrected Model: F_(23, 483)_ = 3.55, p < 0.001, R^2^ = 0.42): age group (F_(2, 488)_ = 93.02, p < 0.001), smoking (F_(1, 488)_ = 13.83, p < 0.001), frequency of daily tooth brushing (F_(3, 488)_ = 8.26, p < 0.001), and tremor (F_(3, 488)_ = 6.53, p < 0.001) (Table2). In other words, older age, smoking, less frequent tooth brushing, and a greater degree of tremor were significantly associated with a higher DMFT score. On the other hand, daily intake of sweets showed a trend-level effect (F_(1, 488)_ = 3.62, p = 0.058) (Table 2).

**Table 2:** DMFT score in association with clinical characteristics

**Characteristics**	**Values (mean ± SD)**	**Statistics**
Age group		F_(2, 488)_ = 93.02, p < 0.001
< 40 years (N =75)	10.7 ± 6.2 ^a^	p < 0.001 vs. 40–59 years; p < 0.001 vs. ≥ 60 years
40 - 59 years (N = 221)	18.8 ± 7.0 ^b^	p < 0.001 vs. < 40 years; p < 0.001 vs. ≥ 60 years
≥ 60 years (N = 227)	23.5 ± 6.3 ^c^	p < 0.001 vs. < 40 years; p < 0.001 vs. 40–59 years
Smoking		F_(1, 488)_ = 13.83, p < 0.001
Yes (N = 172)	21.1 ± 7.4	N.A.
No (N = 351)	19.0 ± 8.0	N.A.
Frequency of daily tooth brushing		F_(3, 488)_ = 8.26, p < 0.001
< Once/day (N = 136)	23.5 ± 7.2 ^d^	p < 0.001 vs. once daily; p < 0.001 vs. twice daily
Once/day (N = 155)	19.4 ± 7.6 ^e^	p < 0.001 vs. no daily tooth brushing
Twice/day (N = 99)	17.6 ± 7.8 ^f^	p < 0.001 vs. no daily tooth brushing
≥ Three times/day (N = 133)	17.7 ± 7.4 ^g^	p < 0.001 vs. no daily tooth brushing
The UKU Tremor subscore		F_(3, 488)_ = 6.53, p < 0.001
0 (N = 294)	18.1 ± 7.8 ^h^	p = 0.001 vs. score 1; p < 0.001 vs. score 2; p < 0.001 vs. score 3
1 (N = 166)	20.6 ± 7.8 ^i^	p = 0.001 vs. score 0; p < 0.001 vs. score 2; p = 0.005 vs. score 3
2 (N = 36)	25.1 ± 4.4 ^j^	p < 0.001 vs. score 0; p < 0.001 vs. score 1
3 (N = 23)	25.1 ± 4.9 ^k^	p < 0.001 vs. score 0; p = 0.005 vs. score 1
Daily intake of sweets		F_(1, 488)_ = 3.62, p = 0.058
Yes (N = 363)	20.1 ± 7.9	N.A.
No (N = 160)	18.7 ± 7.7	N.A.

SD, standard deviation; the DMFT, the Decayed-Missing-Filled Teeth, N.A., not available.

A univariate general linear model: corrected Model: F_(23, 483)_ = 3.55, p < 0.001, R^2^ = 0.42.

Pair wise comparisons were performed by the Tukey-Kramer HSD (honestly significant difference) when the univariate general linear model showed a significant effect of any subgroup/category-of-interest on the DMFT index.

## Discussion

In this study, we found that older age, smoking, less frequent tooth brushing and a greater degree of tremor were associated with a higher DMFT score. While it is well acknowledged that schizophrenia patients often present co-morbidity such as metabolic derangements [3,23], the clinical relevance of their dental hygiene is still under-recognized [4]. It is self-evident that poor dental condition could lead to insufficient digestion; moreover, dental decay has been shown to be significantly associated with aspiration pneumonia [24]. Furthermore, de Oliveira et al. demonstrated an association between poorer oral hygiene and a higher risk of cardiovascular disease in the general population in Scotland (N = 11,869) [25]. In addition, poor dental health itself is associated with a lower quality of life as such patients no longer fully enjoy meals [26-28]. These findings emphasize the need for attention to dental check-ups for patients with schizophrenia. In fact, as Ponizovsky et al. demonstrated that regular dental examinations and treatment for inpatients in psychiatric hospitals substantially improved the dental health of this population [29]; consequently, the implementation of on-site dental services needs to be considered.

One of the novel findings in this cross-sectional study is a close association between more severe tremor and a poorer dental condition. This finding seems attributable to the fact that tremor is very likely to result in impaired fine motor movement, which would be expected to hamper smooth tooth brushing that consists of various elaborate movements. Almost all of schizophrenia patients are receiving antipsychotic medications that have a potential to cause parkinsonian symptoms [30]. Therefore, it is critically important to try to find the lowest possible therapeutic dose of antipsychotics to maintain a patient’s fine motor function that would be needed for smooth brushing skills although antipsychotic dose-reduction clearly needs thorough caution in light of potential clinical worsening [31].

More than half the patients brushed their teeth as few times as once or less a day in the present study. Furthermore, as expected, these patients showed higher DMFT scores than those who more frequently brushed their teeth. Poor dental hygiene has been found to be associated with changes in life-style and preferences that were frequently seen in patients with schizophrenia [32-35]. Previous findings have demonstrated a lack of sufficient motivation in self-care in patients with schizophrenia [12,36,37]. For example, Jovanovic et al. investigated behaviors and interests associated with dental care in 372 psychiatric inpatients and found that, when compared to healthy people, they visited a dentist less frequently, brushed their tooth for shorter periods and less often, and failed to acknowledge the adverse effects of oral diseases on their general health condition [36]. These findings are compatible with our results and highlight the importance of regular tooth brushing by patients or a reminder to do so given by their caregivers. In addition, a high smoking rate is another concern in patients with schizophrenia [38], which was also true for our sample (32.9 %). In the present survey, there was a positive association between smoking and the DMFT score; this is a consistent finding in the literature. Smoking can contribute to poor dental health as it is associated with an increased pocket depth, loss of periodontal attachment and a higher rate of tooth loss [39]. Thus, it should be noted that smoking not only causes a variety of serious physical illnesses, but it also has the potential to result in poor oral-dental hygiene. These findings are particularly clinically relevant in patients with schizophrenia in light of their high smoking rate.

The results of our study must be interpreted in light of a number of limitations. First, the R squared value of 0.42 indicates that approximately half the variability is still not fully explained by the variables that are contained in our model. It would have been ideal to evaluate other clinical characteristics such as financial condition, nutritional status, and brushing technique that are likely to influence a patient’s dental condition. Second, psychotropic drugs were not included in the models in this study although their potential overall impacts on dental condition such as dry mouth and tremor were taken into consideration. Considering that psychotropic regimens are very unlikely to be constant over years [40], this important issue clearly warrants further investigations in a longitudinal fashion. Third, a possibility of selection bias cannot entirely be rejected. Patients who did not agree to take part in this survey were more likely to be reluctant to have a dental check-up because of their poor dental health that they were already aware of. However, even if this holds true, the actual DMFT score in patients with schizophrenia would be worse than the results of this study, which would further emphasize the need for dental care in this population. Fourth, some variables would have been better to be addressed longitudinally. For instance, only current smoking status was evaluated, which precludes any speculation on the plausible cumulative dose–response analysis. Likewise, tremor is expected to take some time to finally translate into observable changes in the teeth. Fifth, we approached only inpatients with schizophrenia in the units whose consultant psychiatrists agreed to this survey; therefore, the sample did not always represent the general population with schizophrenia. Finally, due to the nature of the cross-sectional study design, a causal relationship between clinical characteristics associated with a higher DMFT score and a poor dental condition cannot be unequivocally established. This needs to be addressed in prospective trials.

## Conclusions

The present study found that older age, smoking, severe tremor, and less frequent tooth brushing were negatively related with dental condition in patients with schizophrenia. Given that poor dental hygiene has been reported to be associated with an increased risk of severe physical complications [9,41], physicians should be aware of patients’ dental status, especially for aged patients with schizophrenia who smoke. In addition, in real-world clinical settings where a dentist is unavailable, a simple macroscopic dental check-up that could be feasibly performed by physicians with 1–2 days training may be useful. Furthermore, for schizophrenia patients who present with tremor, it is critically important to try to find the lowest possible antipsychotic dose to maintain their fine motor function for better smooth brushing skills. It may also be advisable for caregivers to encourage and help patients with schizophrenia to perform tooth brushing more frequently, at the very least once, in order to prevent a worsening in their dental condition.

## Competing interests

Dr. Uchida has received grants from Pfizer, speaker’s honoraria from Otsuka Pharmaceutical, Janssen Pharmaceutical, and Shionogi, and manuscript fees from Dainippon Sumitomo Pharma within the past 5 years. Dr. Suzuki has received grants from Kanae Foundation and Mochida Memorial Foundation, and manuscript fees form Dainippon Sumitomo Pharma and Kyowa Hakko Kirin within the past 5 years. Dr. Watanabe has received grants, or consultant fees from Dainippon Sumitomo Pharma, Eli Lilly, GlaxoSmithKline, Janssen Pharmaceutical, and Pfizer, and received speaker’s honoraria from Astellas Pharma, Dainippon Sumitomo Pharma, Eli Lilly, GlaxoSmithKline, Janssen Pharmaceutical, Meiji, Otsuka Pharmaceutical, Pfizer, and Yoshitomiyakuhin within the past 5 years. Dr. Takeuchi has received speaker’s honoraria or manuscript fees from Dainippon Sumitomo Pharma, Eli Lilly, GlaxoSmithKline, Janssen Pharmaceutical, and Otsuka Pharmaceutical within the past 5 years. Dr. Mimura has received grants, or consultant fees from Eisai, Astellas Pharma, GlaxoSmithKline and Meiji, and received speaker’s honoraria from Astellas Pharma, Dainippon Sumitomo Pharma, Eli Lilly, GlaxoSmithKline, Janssen Pharmaceutical, Meiji, Otsuka Pharmaceutical, Pfizer, and Yoshitomiyakuhin within the past 5 years. Other authors have nothing to disclose.

## Authors’ contributions

All authors contributed to and have approved the design and the protocol of the study and the literature searches. HT and HU managed the collection of the data and analyses. HT wrote the first draft of the manuscript, and all authors contributed to and have approved the final manuscript.

## Previous presentation

Some of the data were presented at the European Conference on Schizophrenia Research, Berlin, Germany, September 29, 2011.

## Pre-publication history

The pre-publication history for this paper can be accessed here:

http://www.biomedcentral.com/1472-6831/12/32/prepub
